# Characterization and resuscitation of ‘non-culturable’ cells of *Legionella pneumophila*

**DOI:** 10.1186/1471-2180-14-3

**Published:** 2014-01-02

**Authors:** Adrien Ducret, Maïalène Chabalier, Sam Dukan

**Affiliations:** 1 Aix Marseille Université, Laboratoire de Chimie Bactérienne (UMR7283), Institut de Microbiologie de la Méditerranée - CNRS, 31, Chemin Joseph Aiguier, 13402 Marseille, France; 2 Present address: Department of Biology, Indiana University, 1001 East 3rd Street, Bloomington, IN 47405 USA

## Abstract

**Background:**

*Legionella pneumophila* is a waterborne pathogen responsible for Legionnaires’ disease, an infection which can lead to potentially fatal pneumonia. After disinfection, *L. pneumophila* has been detected, like many other bacteria, in a “viable but non culturable” state (VBNC). The physiological significance of the VBNC state is unclear and controversial: it could be an adaptive response favoring long-term survival; or the consequence of cellular deterioration which, despite maintenance of certain features of viable cells, leads to death; or an injured state leading to an artificial loss of culturability during the plating procedure. VBNC cells have been found to be resuscitated by contact with *amoebae.*

**Results:**

We used quantitative microscopic analysis, to investigate this “resuscitation” phenomenon in *L. pneumophila* in a model involving amending solid plating media with ROS scavengers (pyruvate or glutamate), and co-culture with *amoebae*. Our results suggest that the restoration observed in the presence of pyruvate and glutamate may be mostly due to the capacity of these molecules to help the injured cells to recover after a stress. We report evidence that this extracellular signal leads to a transition from a not-culturable form to a culturable form of *L. pneumophila*, providing a technique for recovering virulent and previously uncultivated forms of *L. pneumophila*.

**Conclusion:**

These new media could be used to reduce the risk of underestimation of counts of virulent of *L. pneumophila* cells in environmental samples.

## Background

*Legionella pneumophila* is a waterborne pathogen that can survive in a wide range of environmental conditions [1]. It is the etiological agent of Legionnaires’ disease, which can progress to fatal pneumonia [2]. Transmission between individuals is not observed, but aerosol transmission from the aquatic environment to humans is now well documented [1-4]. After inhalation and dissemination into the lungs, *L. pneumophila* invades alveolar macrophages where it multiplies intracellularly. The presumed natural reservoir for this pathogen in the environment is amoebae, where *L. pneumophila* can invade and replicate [5,6]. *L. pneumophila* switches between two forms —a non-motile, thin-walled replicative form and a motile, thick-walled transmissive form— allowing it to survive in the face of environmental fluctuations [7,8]. These two phases of the *L. pneumophila* life cycle are reciprocal and the transition between them is triggered by the amount of available nutrients [9-11]. In favorable conditions, transmissive traits are repressed, enabling *L. pneumophila* to replicate profusely. By contrast, when nutrients become limiting, *L. pneumophila* cells stop replicating and express virulence factors that mediate survival and dispersal in the environment. By comparison with the replicative form, the transmissive form is characterized by cell motility, osmotic resistance, sodium sensitivity, cytotoxicity and the ability to avoid phagosome-lysosome fusion [10]. Under certain conditions, transmissive *L. pneumophila* develop into ‘mature intracellular forms’ that can persist in the environment [12].

Prevention and eradication of *L. pneumophila* contamination of man-made water systems is required to avoid and control legionellosis outbreaks. For this purpose, a large range of physical, thermal and chemical methods are used, including metal ions (copper and silver), UV light, and oxidizing and non-oxidizing agents [13,14]. *L. pneumophila* has been detected in a “viable but non culturable” (VBNC) state immediately after such disinfection [15-19]; the VBNC state is a physiological state in which bacteria cannot grow on standard growth media but retain certain features of viable cells, such as cellular integrity, metabolic activity or virulence [20]. The physiological significance of the VBNC state is unclear and controversial: it could be an adaptive response favoring long-term survival under adverse conditions [21,22] (referred to hereafter as adaptive-VBNC or A-VBNC cells) or the consequence of cellular damage which despite the maintenance of some features of viable cells leads to death (damaged VBNC or D-VBNC cells) [23-25].

It has been reported that apparently dead cells could be restored to viability on agar plates supplemented with compounds that degrade or block the formation of reactive oxygen species (ROS) [26-35]. Various stresses, including starvation, hypochlorous acid (HOCl) and heat shock, may leave cells in a vulnerable physiological state (injured state) in which atmospheric oxygen, during the plating procedure, may amplify cellular damage leading to an artifactual loss of culturability [26-35]. In other words, cells detected as VBNC may be A-VBNC cells or D-VBNC cells or cells in an injured state. The existence of A-VBNC or injured pathogen cells is a public health concern since they would not be detected as possible sources of infection, and may nevertheless retain their pathogenicity. For instance, samples containing *L. pneumophila* have been exposed to starvation, monochloramine or HOCl, and as a result contained no detectable culturable cells; however, *L. pneumophila* was resuscitated by contact with *amoebae*[16,18,36,37], suggesting that non-culturable *L. pneumophila* cells were still able to invade *amoebae* and replicate*.* However, this “resuscitation” phenomenon may simply reflect the presence of injured or A-VBNC cells.

We used quantitative microscopic analysis, and a model system involving amending solid plating media with ROS scavengers, and co-culture with *amoebae*, to investigate this “resuscitation” phenomenon. We show that including the ROS scavengers, pyruvate and glutamate, in standard medium (BCYE) may reduce underestimation of the counts of pathogenic and not-culturable forms of *L. pneumophila* in environmental samples. Our findings indicate that the restoration observed in the presence of pyruvate and glutamate may be largely due to these compounds facilitating the recovery of injured cells after a stress.

## Results

### Two sub-populations of viable *L. pneumophila* cells were observed before and after a HOCl treatment

**Figure 1:**
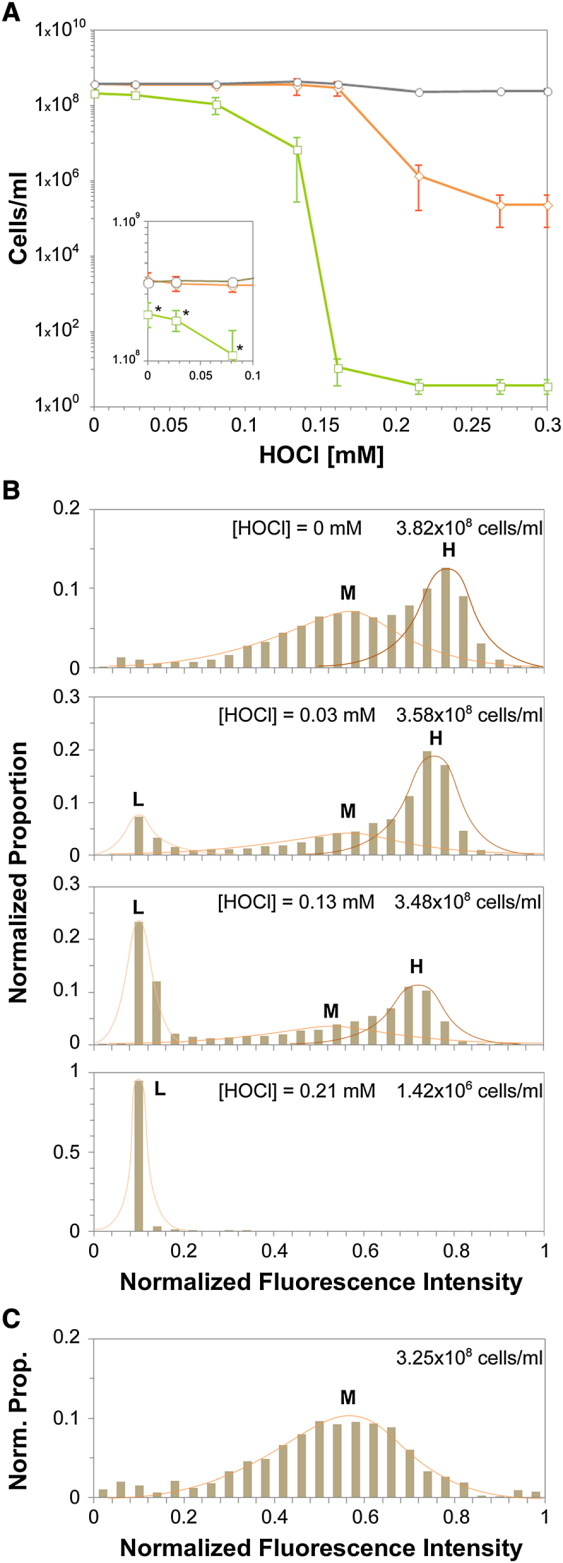
**Culturability and viability of *L. pneumophila Philadelphia *cells harvested at the beginning of stationary phase, before and after HOCl treatment. (A)** Number of culturable cells as assessed on the standard medium (□), total number of cells detected using DAPI procedure (○), and viable cells detected using the CV6 procedure (◊) as a function of HOCl concentration (mM). The values reported are the means of three independent experiments (Errors bars = SD). Inset shows a close-up of the part of the plot corresponding to HOCl concentrations lower than 0.1 mM. Stars indicate that the number of culturable cells was significantly lower (p < 0.05) than the total number of cells. **(B)** Distribution of the normalized fluorescence intensity of the viable cells detected using the CV6 procedure as a function of HOCl concentration. Subpopulations were named according to their relative fluorescence intensity: L (*Low*), M (*Medium*) and H (*High*). **(C)** Distribution of the normalized fluorescence intensity of viable cells detected using the CV6 procedure in samples harvested from a culture in the exponential growth phase. For each condition, at least 3000 cells were analyzed. Similar results were obtained in two other independent experiments.

To confirm previous detection of VBNC cells (A-VBNC cells D-VBNC cells plus injured cells) of *L. pneumophila*[15-19], the culturability and the viability of a suspension of *L. pneumophila* cells harvested at the beginning of stationary phase was investigated before and after a HOCl treatment (see Methods). Culturability was determined on the standard medium (BCYE) and cell viability was assessed using a ChemChrome V6 Kit (CV6). This assay kit is widely used to discriminate metabolically active cells (which become fluorescent) from dead cells (which do not fluoresce), and has been used to detect VBNC *L. pneumophila* cells [18,38,39]. As expected, the number of culturable and viable cells decreased as the HOCl concentration increased, but the total number of cells observed did not change significantly (Figure 1A). Viable counts determined by CV6 were significantly higher (p < 0.05) than CFU counts in all samples, indicating the presence of VBNC cells even in samples not treated with HOCl (Figure 1A).

CV6 is a fluorogenic ester which is converted to free fluorescein by cytoplasmic esterases. Since the concentration of fluorescent fluorescein trapped in metabolically active cells increases over the time as a function of esterase activity, the level of fluorescence is a marker of the specific metabolic activity at the single-cell level. We therefore followed the distribution of fluorescence in the viable cells before and after the HOCl treatment (Figure 1B). The distribution of the fluorescence intensity was not uniform: there were distinct peaks of cell numbers at certain intensities suggesting that the population of cells was composed of distinct sub-populations of viable cells with different degrees of metabolic activity. Two sub-populations with normal and overlapping distributions were observed even before the HOCl treatment: a sub-population centered to the average value of fluorescence intensity (1.52 × 10^8^ cells.ml^-1^), albeit showing some diversity in values, and subsequently referred as subpopulation M (medium), and a sub-population with high, and more similar, values of the fluorescence intensity (1.55 × 10^8^ cells.ml^-1^), referred to as subpopulation H (high). When this analysis was repeated with cells were harvested during exponential growth, only one of these two subpopulations, subpopulation M was observed (Figure 1C).

At very low HOCl concentration (0.03 mM; 52% of culturable cells; 95% of viable cells), subpopulation H was not affected (1.51 × 10^8^ cells.ml^-1^) but subpopulation M was substantially reduced (0.73 × 10^8^ cells.ml^-1^) with the concomitant apparition of a new subpopulation (0.71 × 10^8^ cells.ml^-1^) characterized by a very low level of fluorescence (subpopulation L). At HOCl concentrations associated with a decrease in the CFU counts (0.13 mM; 1.6% of culturable cells; 81% of viable cells), subpopulation H was again not substantially affected (1.11 × 10^8^ cells.ml^-1^) whereas the subpopulation M was almost undetectable, subpopulation L was large (2.58 × 10^8^ cells.ml^-1^). At the highest concentration of HOCl (0.21 mM; 1.6 × 10^-6^% of culturable cells; 0.6% of viable cells), neither subpopulation M nor H was detected, and only subpopulation L was observed.

These findings indicate that there are at least two subpopulations of metabolically active cells in *L. pneumophila* cultures harvested at the beginning of the stationary phase. Interestingly, these two subpopulations were affected differently by treatment with the biocide HOCl: the most metabolically active subpopulation (H) was more resistant than the less active subpopulation (M), and appeared to be the majority subpopulation among the VBNC cells observed after moderate HOCl treatment (0.03-0.13 mM).

### The culturability of a VBNC cell subpopulation on standard medium was restored by the presence of pyruvate and/or glutamate

The detection of VBNC cells upon treatment with HOCl displaying metabolic activity close to the level observed in absence of treatment (population H), suggest that these cells were still active but unable to form colonies on agar plates. There have been numerous reports that apparently dead cells (injured cells) could be reactivated by inclusion of ROS scavengers in agar plates [26-35]. We therefore added various concentrations of compounds that degrade or block the formation of ROS to standard medium (BCYE) (Table 1). *L. pneumophila* cultures were treated with 0.21 mM HOCl and plated on the various media. The ratio of cells counts on supplemented medium to that on standard medium was calculated as a measure of recovery. This ratio was higher than 1 only for pyruvate and glutamate: these compounds thus promoted the recovery of presumably injured cells. The highest recovery ratio was observed in presence of 0.5% (w/w) pyruvate: the culturable *L. pneumophila* cell count was 150 times higher on supplemented BCYE (BCYES) than the standard medium (BCYE). The cell counts on plates containing both pyruvate (0.5% w/w) and glutamate (1% w/w) were 1000 times higher than on standard medium suggesting a strong synergetic effect (Table 1).

**Table 1:** Restoration ratio in presence of supplements

**Supplements**	**Restoration ratio**
Sodium Pyruvate [%]	
0.1	1.1 ± *0.2*
0.5	**154.8** ± *11*
1	**62.5** ± *25*
Glutamate [%]	
0.1	0.7 ± *0.6*
0.5	**3.0** ± *0.2*
1	**3.9** ± *0.3*
α-Ketoglutaric acid [%]	
0.05	0.2 ± *0.2*
0.25	0.1 ± *0.1*
0.5	0.0 ± *0.0*
Propyl gallate [%]	
0.005	1.1 ± *0.1*
0.025	1.3 ± *0.3*
0.05	1.1 ± *0.5*
Ethoxyquin [%]	
0.05	0.1 ± *0.2*
0.25	0.1 ± *0.1*
0.5	0.0 ± *0.0*
DMSO [%]	
0.005	1.0 ± *0.04*
0.025	0.9 ± *0.03*
0.05	0.8 ± *0.06*
Ascorbic Acid [%]	
0.005	0.9 ± *0.15*
0.025	0.9 ± *0.2*
0.05	0.0 ± *0.0*
3,3′ Thiodipropionic Acid [%]	
0.005	1.0 ± *0.08*
0.025	1.0 ± *0.07*
0.05	0.0 ± *0.00*
Glutamate [%] + 0,5% Sodium Pyruvate	
0.1	**160.0** ± *21*
0.5	**450** ± *91*
1	**884** ± *117*

Restoration ratio greater than 1.5 are displayed in Bold. Standard deviation are displayed in italic.

**Figure 2:**
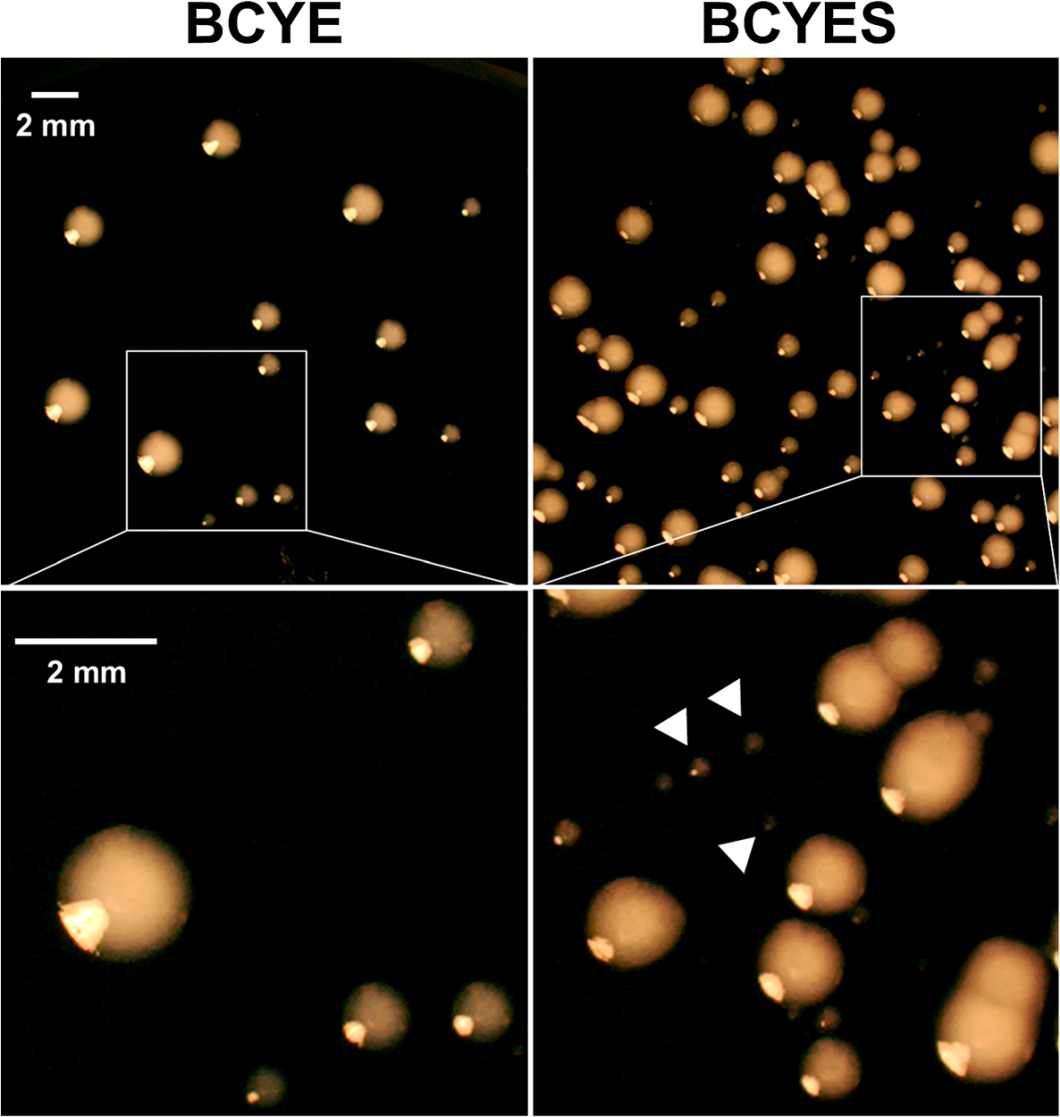
**Images of the colonies observed on the standard medium (BCYE) and the standard medium supplemented with pyruvate (0.1%) and glutamate (0.5%) (BCYES).** Representative results from one of two independent experiments are shown.

Careful examination of BCYES plates revealed two types of colonies: colonies with diameters similar to those on standard medium (3–4 mm) and colonies with very small diameters (< 1 mm) (Figure 2). Small colonies are generally indicative of lower growth rates and/or longer latency period, this observation suggests that the restored population was composed of at least two subpopulations with two different levels of physiological activity.

**Figure 3:**
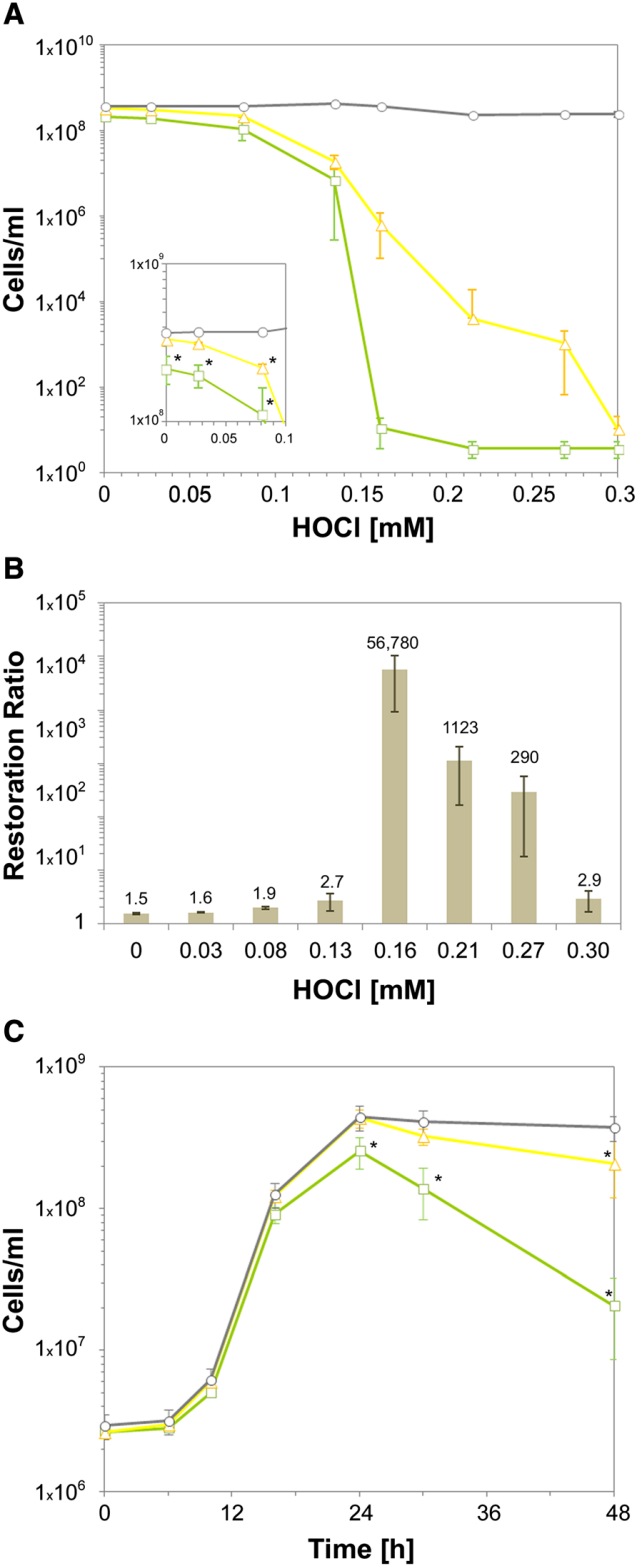
**Restoration of the culturability of *L. pneumophila Philadelphia *cells on supplemented medium (BCYES). (A)** Number of culturable cells observed on standard medium (□), total cells (○) and culturable cells observed on the supplemented medium (∆) as a function of HOCl concentration (mM). The results reported are means of three independent experiments. Inset shows a magnification of the region of the plots corresponding to HOCl concentrations lower than 0.1 mM. Stars indicate that the number of culturable cells was significantly lower (p < 0.05) than the total number of cells. **(B)** Restoration ratio (Number of culturable *L. pneumophila* cells on supplemented medium divided by that on standard medium) as a function of HOCl concentration. The restoration ratio is given above each bar. **(C)** Number of culturable cells as assessed on the standard medium (□), total cells (○) and culturable cells as assessed on the supplemented medium (∆) as a function of time (h) for cultures in the liquid standard medium (YEC) at 37°C. The results reported are means of three independent experiments (Errors bars = SD). Stars indicate that the number of culturable cells is significantly lower (p < 0.05) than the total number of cells.

We evaluated the degree of restoration following treatment with, and without, various concentrations of HOCl (Figure 3A & B). The recovery ratio increased from 1.6 to more than 50,000 as the HOCl concentration increased from 0.03 to 0.16 mM, and then dropped to 2.9 for the highest concentration of HOCl. Interestingly, even in absence of HOCl treatment, a subpopulation of cells could be restored on the supplemented medium.

We assessed the degree of restoration during cell growth (Figure 3C). The recovery ratio increased with the time of culture: the restored population was small for samples collected during exponential growth, but was the major subpopulation for samples collected during late stationary phase.

These results show that the culturability on standard medium of a subpopulation of VBNC cells was substantially enhanced by the presence of pyruvate and/or glutamate. Two types of colonies were observed on the supplemented medium, suggesting that the restored population was made up of two subpopulations with different levels of physiological activity.

### Apparently injured cells are able to invade and replicate in *Amoeba*

The VBNC *L. pneumophila* cells described by several research groups can be resuscitated when co-cultured with *Amoebae*[16,18,36,40]. We tested whether this apparently injured subpopulation was able to invade, and replicate in, *Amoebae*. This subpopulation can only be detected by appropriate plating procedures, we were unable to specifically sort this subpopulation and test its specific virulence.

**Figure 4:**
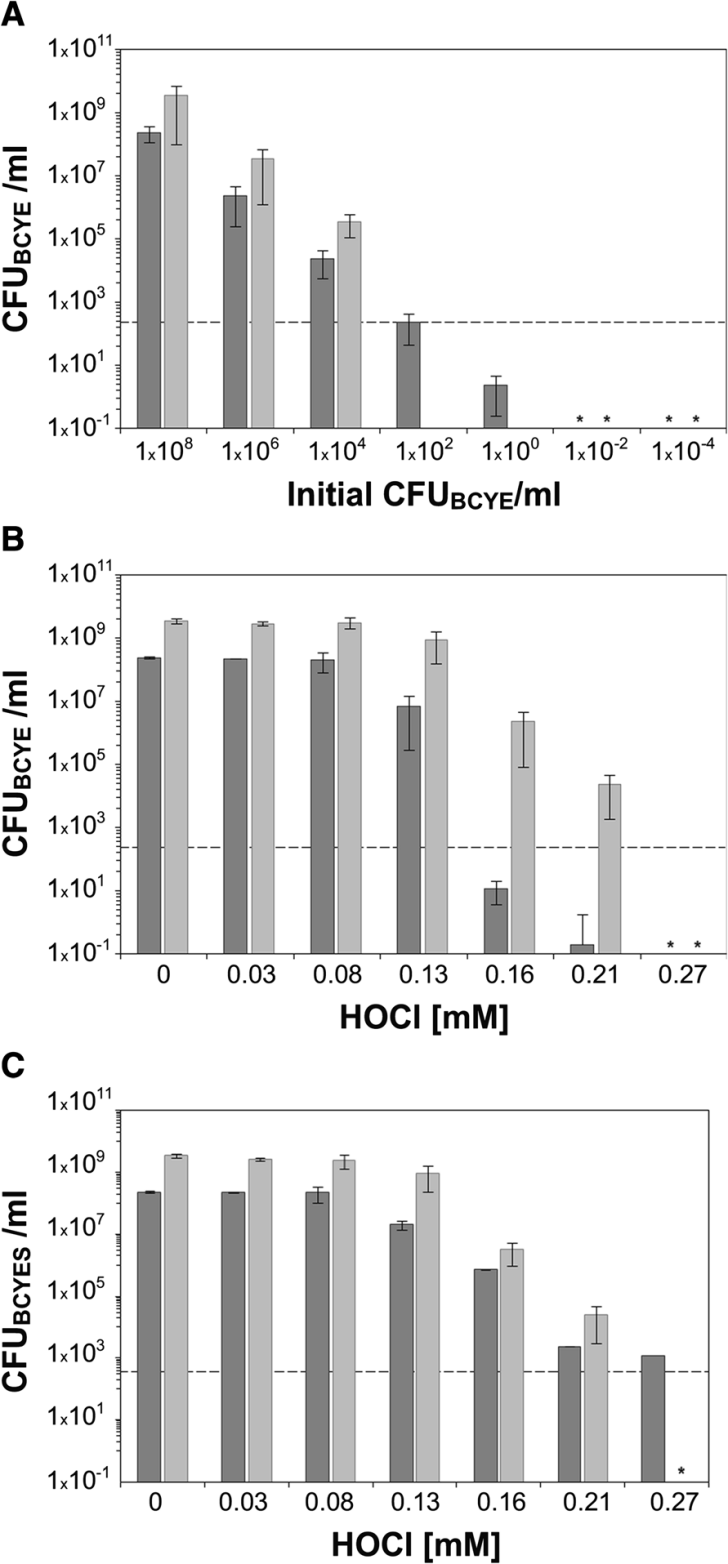
**Proliferation of *Legionella *cells in co-culture with *A. castellanii. *(A)** Proliferation of serially diluted culturable cells is represented as a function of the initial number of CFU as assessed on the standard medium (BCYE). **(B & C)** Proliferation of cell suspensions exposed to various concentrations of HOCl based on the initial number of CFU as assessed on the standard medium (BCYE) **(B)** or the supplemented medium (BCYES) **(C)**. Dark bars represent the initial number of CFU as assessed on the standard medium or the supplemented medium (BCYES). Gray bars represent the number of CFU as assessed on the BCYE medium after co-incubation with axenic culture of *A. castellanii.* The values reported are means for duplicate samples in three independent experiments. Error bars indicate SD and asterisks values below the detection limit (<0.1 CFU.ml^-1^).

To overcome this difficulty, we first identified the minimal number of culturable cells allowing proliferation of *L. pneumophila* when co-cultured with *Amoebae*. Culturable cells were diluted in a suspension of 3.5 10^8^ heat-killed legionella cells.ml^-1^ such that there were similar numbers of cells in each sample tested. Initial CFU counts were assessed on standard medium and these mixed cell suspensions were then co-incubated with axenic cultures of *A. castellanii.* The proliferation of serially dilutions of these cultures correlated with the initial number of culturable cells: 50 to 100 times more culturable cells were observed after co-culture (Figure 4A). Moreover, no proliferation was observed for suspensions containing less than 10^2^ CFU.ml^-1^ before co-culture. This indicates that *L. pneumophila* proliferation in contact with *A. castellanii* was a function of the initial number of culturable cells and at least 10^2^ CFU.ml^-1^ is required for proliferation to be detected in our conditions.

Then, we co-incubated HOCl-treated suspensions of *L. pneumophila* with axenic cultures of *A. castellanii.* Initial CFU counts were assessed both on standard and supplemented (BCYES) media. When CFU counts were assessed on standard medium, *L. pneumophila* proliferation was observed with several suspensions of *L. pneumophila* containing less than 10^2^ CFU.ml^-1^ and also the proliferation rates (1000 to 10000) were higher than those observed in calibrated experiments (50 to 100) (Figure 4B). This difference with the results of the calibrated proliferation experiment (Figure 4A) suggests existence of a subpopulation of cells that were not culturable on the standard medium but that were nevertheless able to infect *A. castellanii* and then grow. Part of the proliferation in this model system could therefore be interpreted in as a “resuscitation”. The initial number of culturable cells assessed from CFU counts on BCYES was always higher than that observed on the standard medium (Figure 4C). In this condition, no proliferation of *L. pneumophila* was observed after co-culture for suspensions containing less than 10^2^ CFU.ml^-1^ and the proliferation rates were similar to those observed in calibrated experiments (50 to 100; Figure 4A). Thus, after HOCl treatment, proliferation was a function of the initial number of culturable cells assessed on the BCYES medium but not on the standard medium (BCYE). In other terms, proliferation, and as a consequence, the phenomena of resuscitation observed on the standard medium corresponded, at least in large part, to the subpopulation of cell restored (the injured cells) on the BCYES medium.

## Discussion

Before the advent of single-cell based analytical methods, researchers worked mostly with pure cultures assuming that the behavior of each single cell in a population is consistent with the average behavior of all cells. However, it has been demonstrated that cell behavior in a bacterial population is divergent even under identical micro-environmental conditions. Complex phenomenon such as stochasticity in gene expression [41], asymmetrical aging [42], asymmetrical division [43], bi-stability [44] and cell differentiation [7] can lead to the formation of sub-populations with different cellular physiologies and/or morphologies. Unfortunately, the link between the cellular physiology and culturability of each sub-population is not always clear, and as a consequence the characterization of VBNC cells of some organisms is complex.

VBNC *L. pneumophila* cells have been observed by many groups [16,18,36,37,40] but the mechanisms leading to this physiological state remain unknown. It could be part of an adaptive response (A-VBNC cells), and/or a consequence of cellular deterioration (D-VBNC cells) and/or a consequence of cell death during the plating procedure (injured cells), all leading to the inability of the *L. pneumophila* cell affected to form a colony. In our study, we assessed the viability of *L. pneumophila* at the single-cell level using the CV6 procedure. By using high efficiency cell-counting procedures (n > =3000), VBNC cells were detected after, but also in the absence of, biocide treatment. Interestingly, two subpopulations of cells with different levels of metabolic activity were identified among VBNC cells. These two populations displayed different resistance to the biocide treatment, suggesting that they have different physiological characteristics. We also found that pyruvate and/or glutamate were able to restore the culturability to a large proportion of the non-culturable cells observed both after, but also in the absence of, biocide treatment. Importantly, we demonstrate that the restored population was able to invade amoeba and then replicate, and that this was responsible for the “resuscitation” phenomenon. These observations strongly suggest that a suspension of *L. pneumophila* cells harvested at the beginning of stationary phase is composed of different sub-populations, with different physiological characteristics, susceptibility to stress, culturability and ability to be restored by pyruvate and/or glutamate.

It remains unclear exactly how pyruvate and glutamate promote restoration. Pyruvate is an antioxidant that neutralizes or prevents the formation of ROS in rich medium [26,27,29,32,34]. When pyruvate is converted to alanine, glutamate is concomitantly converted to α-ketoglutarate [45], a substrate already present in the medium used for *L. pneumophila* (BCYE) and also used, for 25 years, in combination with charcoal to support the growth of *Legionella* species [46]. Keto acids prevent the toxic effects of light by inhibiting superoxide production and inhibit the rate of cysteine oxidation, an amino acid present in excess in the medium because of the cysteine auxotrophy of *L. pneumophila* species [46]. The presence of glutamate as well as pyruvate may lead to the formation of antioxidant compounds that directly or indirectly help a subpopulation of injured cells to recover during the plating procedure [26-35]. However, when other antioxidant compounds, including ascorbic acid, propyl gallate or α-ketoglutarate, were added to the standard medium, they failed to significantly restore the culturability of non-culturable *L. pneumophila* cells (Table 1). Therefore, the action of pyruvate and glutamate may not be associated with their antioxidant properties. Pyruvate and glutamate may be involved in the complex life cycle of *L. pneumophila*. Although signal molecules that trigger *L. pneumophila* differentiation from the replicative to the non-replicative and transmissive form have been thoroughly studied [7,9-11], the signal triggering the reciprocal transition from the transmissive to the replicative form remains unknown. Several observations imply that amino acids are the primary signals driving differentiation from the transmissive to the replicative form of *L. pneumophila,* and it is therefore plausible that glutamate, one of the most abundant amino acids, might stimulate this differentiation [7]. Also, pyruvate can be converted into carbohydrates via gluconeogenesis, to the amino acid alanine, to fatty acids or to energy through acetyl-CoA. Thus, a combination of the actions of glutamate, alanine and perturbations in fatty acid metabolism [9] may act as an integrated signal to trigger the transition from the virulent to the replicative form of *L. pneumophila*.

## Conclusion

Our results suggest that the restoration of non-culturable *L. pneumophila* observed in presence of pyruvate and glutamate may be a consequence of their ability to help the injured cells to recover after a stress. However, we cannot exclude the possibility that pyruvate and glutamate also drive differentiation from the transmissive to the replicative form of *L. pneumophila*. Moreover, we report evidence that this extracellular signal leads to the transition from a not-culturable form to a culturable form of *L. pneumophila*, providing a means for recovering virulent and previously uncultivated forms of *L. pneumophila*. These new media may be valuable for reducing the risks associated with underestimation of virulent cell counts of *L. pneumophila* in environmental samples.

## Methods

### *S*train and growth conditions

CIP 103854 T, *L. pneumophila* Philadelphia was used. Bacteria were frozen at −80°C until use. Bacteria were grown on buffered charcoal yeast extract (BCYE) agar plates supplemented with L-cysteine, ferric pyrophosphate and α-ketoglutarate (OXOID) for 72 h at 37°C. From solid medium, strains were grown aerobically in a rotary shaker at 37°C and 160 rpm in YEC, a liquid yeast extract medium supplemented with L-cysteine, ferric pyrophosphate and α-ketoglutarate. Overnight cultures were diluted 100-fold in YEC liquid medium and were allowed to grow for 24 h (to the stationary phase).

### Biocides challenge

Stationary phase *L. pneumophila* cells were harvested from 250 ml cultures and then washed twice with phosphate buffer (pH 7.4) by centrifugation at 5500 × *g* for 10 min at 4°C. Cells were resuspended and diluted in PBS pH 7.4 to an optical density of 0.2 at 600 nm (1 × 10^8^ cells ml^-1^). These cell suspensions (50 ml each) were distributed into 100-ml glass flasks, and fresh HOCl solution (prepared the same day) was added to various concentrations from 0 to 1 mM (≤1 ml). The samples were incubated for 1 h at 37°C in the dark with agitation (160 rpm), and the HOCl was then quenched by the addition of sterile sodium thiosulfate (final concentration 0.4 mM). Culturable bacteria were assayed by plating serial dilutions in PBS on BCYE plates at 37°C. Colonies were counted after 3 days and 10 days of incubation at 37°C.

### Viability staining procedure

Viability of *L. pneumophila* was assessed using the ChemChrome V6 procedure (ChemChrome V6; CV6 – AES-Chemunex, Ivry-sur-seine, France) and the total number of cells was assessed using the DAPI procedure (DAPI Nucleic Acid Stain; Invitrogen). Aliquots of a suspension of 1 × 10^7^ cells ml^-1^were used for staining experiments. Labeling solutions were added to the samples according to the manufacturer’s instructions and incubated at 37°C for 30 min in the dark; they were washed by centrifugation (4500 × *g* for 10 min in PBS pH 7.4) and transferred into 96-well glass-bottom micro plates (Greiner Bio One) previously treated with poly-L-lysine (0.01%). To favor cell adhesion to the wells, the plates were centrifuged at 1000 × *g* for 20 min. For each condition, the number of viable cells and the total number of cells were counted, and the results reported are mean values for three independent wells in which at least 3000 cells were analyzed. The distribution of the normalized fluorescent intensity of cells as detected by microscopy is presented as histograms. The values 0 and 1 represent the maximum and the minimum values, respectively, of fluorescence observed in all tested conditions for each experiment. The proportion of each class were normalized to the number of viable cells. The Mann–Whitney U test was used to assess the significance of differences in viable, total and culturable cell numbers.

### Fluorescence microscopy

Microscopic analyses were performed using the automated and inverted epifluorescence microscope TE2000-E-PFS (Nikon) with the appropriate filter blocks as previously described [47]. For each sample, at least 3000 cells were examined from approximately 100 digital images taken automatically with a motorized stage (Prior Scientific), a CoolSNAP HQ 2 (Roper Scientific) and a 40×/0.75 DLL “Plan-Apochromat” or a 100×/1.4 DIC objective. Metamorph 7.5 (Molecular Devices) or NIS-Element (Nikon) software was used for digital analysis and treatment of the images to extract the number, specific fluorescence intensity and length of stained bacteria. This system allows enumeration and manual differentiation between individually labeled cells, cells in aggregations and/or auto fluorescent particles which can interfere with automated analysis. For the CV6 procedure, cells were scored as viable if their fluorescence intensity was at least 1.5 times greater than the fluorescence background noise. Under our conditions, detection limits for CV6 measurement were 3 × 10^4^ cells ml^-1^.

### Co-culture of *L. pneumophila* and *A. Castellanii*

Axenic cultures of *A. castellanii* (ATCC 30234) were prepared as previously described [48]. Briefly, the *A. castellanii* strain was grown in a 150-cm^2^ cell culture flask in PYG medium (peptone-yeast extract-glucose) at 30°C for 3 days. Monolayers were developed in 24-well tissue culture plates using Page’s amoeba Saline (PAS) for 24 h at 30°C. Aliquots of 1 × 10^6^ amoebae per well in 24-well tissue culture plates were infected with 10 × 1 ml of *L. pneumophila* at 1 × 10^8^ cells ml^-1^ in PAS as described above (MOI 100). The plates were centrifuged at 500 × g for 5 min and incubated for 3 days at 37°C. Then, the monolayer and supernatant were removed and spread on BCYE agar plates. Colonies were counted after 3 days and 10 days of incubation at 37°C.

## Authors’ contribution

Conceived and designed the experiments: AD, SD. Performed the experiments: AD, MC. Analyzed the data: AD, MC, SD. Wrote the paper: AD, SD. All authors read and approved the final manuscript.
